# Genome-wide identification and expression analysis of ethylene responsive factor family transcription factors in *Juglans regia*

**DOI:** 10.7717/peerj.12429

**Published:** 2021-11-19

**Authors:** Tianyu Wang, Xiangqian Gao, Sisi Chen, Dapei Li, Shuwen Chen, Muhong Xie, Zhenggang Xu, Guiyan Yang

**Affiliations:** 1 Laboratory of Walnut Research Center, College of Forestry, Northwest A&F University, Yangling, Shaanxi, China; 2 Key Laboratory of Economic Plant Resources Development and Utilization in Shaanxi Province, College of Forestry, Northwest A&F University, Yangling, Shaanxi, China

**Keywords:** *Juglans regia*, Ethylene response factor, Bioinformatics, Expression analysis

## Abstract

**Background:**

Walnut is an important economic tree species with prominent economic value and ecological functions. However, in recent years, walnuts have become susceptible to drought stress, resulting in a decline in comprehensive benefits. Therefore, it is necessary to identify the regulatory molecular mechanism associated with walnut response to drought. In many plants, ethylene responsive factor (*ERF*) gene family plays important roles in response to biotic and abiotic stress, especial drought. Therefore, the identification and characterisation of walnut *ERF* genes will benefit walnut with regard to the clarification of drought response mechanism as well as the management, production, and quality of plantations.

**Methods:**

‘ERF’ was compared against the walnut transcriptome, and the *JrERF*s with a complete open reading frame (ORF) were identified by ORF Finder. The molecular weights, amino acid residues, and theoretical isoelectric point (pI) were predicted by ExPASy. The distribution of *JrERF*s in chromosome locations was determined based on walnut genome data from NCBI. The intron-exon structures and conserved domains were analysed using Gene Structure Display Server 2.0 and CD-Search, accordingly. Multi-sequence alignment and a phylogenetic tree were constructed by ClustalX2.1 and MEGA7, respectively. The conserved motifs were acquired using MEME. Total RNA was isolated using the cetyltrimethylammonium ammonium bromide (CTAB) method (Yang et al., 2018). Gene expression was determined by using real-time quantitative polymerase chain reaction (qRT-PCR) analysis and calculated according to the 2^−ΔΔCT^ method (Livak & Schmittgen, 2001).

**Results:**

A total of 44 *JrERF*s were identified from the walnut transcriptome, whose ORFs were 450–1,239 bp in length. The molecular weights of the JrERF proteins (consisting 149–412 amino acids) were 16.81–43.71 kDa, with pI ranging from 4.8 (*JrERF11*) to 9.89 (*JrERF03*). The *JrERF*s can be divided into six groups (B1–B6), and among the groups, B6 contained the most number of members. Each JrERF contained 1–6 motifs and each motif comprised 9–50 amino acids. Among the motifs, motif1, motif2, and motif3 were the most abundant. More than 40% of *JrERF*s were up-regulated continuously when subjected to ethephon (ETH), PEG_6000_, and PEG_6000_+ETH treatments. Of all the *JrERFs, JrERF11* showed the highest expression. Therefore, we conclude that walnut *ERF* genes are highly conserved and involved in the regulation of drought response in the presence of ETH. *JrERFs* are possibly important candidate genes for molecular breeding; hence, the findings of this study provides the theoretical basis for further investigation of *ERF* genes in walnut and other species.

## Introduction

*Juglans regia* is an economic tree species and is distributed widely all over the world (Abdallah et al., 2015). In China, walnut has become an important woody oil tree species for Poverty Alleviation and Rural Revitalization (Yang et al., 2019). However, like other plants, the growth and development of walnut is restricted by biotic (such as pests, diseases) and abiotic factors (such as moisture, temperature, and light). These factors cause a sharp reduction in the yield and quality of walnut. When exposed to external stimuli, plants mobilise protective mechanisms to reduce damage through many pathways, including releasing stress signals, adapting to stress stimuli, activating a series of molecular pathways, regulating related gene expression, and physiological responses (Hilker & Schmülling, 2019). For example, WRKY, NAC, MYB, AP2/ERF, and bZIP transcription factor (TF) gene families in *Arabidopsis thaliana* were highly enriched and involved in regulating the expression of 56% of common genes in response to drought and cold stresses (Sharma et al., 2018). *GmWRKY54* transgenic soybean improved drought tolerance through abscisic acid (ABA) and Ca^2+^ signalling pathways (Wei et al., 2019). To enhance tolerance to high temperature stress, the expression of walnut *JrGRAS2* stimulates the transcription activity of heat shock proteins (Yang et al., 2018). Therefore, the identification of important TF families can reveal the stress adaptation mechanism of walnut. It will provide a theoretical basis for adversity-related molecular breeding.

TFs are the key molecules for the regulation of gene expression and exist in all organisms. APETALA2/ethylene responsive factor (AP2/ERF) is one of the TF families in plants. After the first AP2/ERF family TF was isolated and identified from *A. thaliana* in 1994 (Jofuku et al., 1994), it had been reported widely in various plants, such as *Ananas comosus* (Zhang et al., 2021), *Betula platyphylla* (Lv et al., 2020), and *Raphanus sativus* (Karanja et al., 2019*)*. According to the type and number of AP2/ERF conserved domain, AP2/ERF TFs can be divided into the following subfamilies: APETALA2 (AP2), related to ABI3/VP1 (RAV), dehydration-responsive element binding protein (DREB), ERF, and Soloist (Sakuma et al., 2002). AP2 subfamily, containing two highly conservative AP2/ERF domains, is involved in cell growth and differentiation (Luo et al., 2020). RAV subfamily, including one AP2/ERF and one B3 domains, is involved in plant flowering and stress response (Zhao et al., 2017). DREB subfamily has an AP2/ERF domain, whose amino acid 14 and 19 are valine and glutamic acid, respectively. DREB has been found to be positive response genes in low temperature, drought, and ABA signalling pathways (Sarkar et al., 2019). The ERF subfamily has only one AP2/ERF domain, whose amino acid 14 and 19 are alanine and aspartic acid, respectively, which are the sites that distinguish the subfamily of ERF from DREB. ERF subfamily relates with various stimuli, such as hormones, low temperature, drought, salt, and pathogens (Lv et al., 2020). Soloist is an orphan of the AP2/ERF family with only one AP2/ERF domain, whose amino acid motif and gene structure are significantly different from those of other AP2/ERF subfamilies. Soloist is mainly involved in the response to low temperature and the associated signal transduction pathways (Wang et al., 2018).

Among the subfamilies of AP2/ERF, ERF has received the greatest attention because of its broad response to various stresses. For instance, plants with Arabidopsis *ERF96* overexpression displayed enhanced resistance to necrotrophic pathogens, which included the fungus *Botrytis cinerea* and the bacterium *Pectobacterium carotovorum* (Fröschel et al., 2019). Ectopic expression of *Phaseolus vulgaris ERF35* in tobacco promoted salt stress tolerance (Kavas et al., 2020). Apple *ERF38* played positive role in drought tolerance relating to anthocyanin biosynthesis (An et al., 2020). *Solanum lycopersicum ERFB3* could respond to cold, heat, and flooding and plays a role in the layout of stress symptoms under cold stress (Klay et al., 2014). Especially in the regulation of drought tolerance, ERF has attracted much attention. For example, the overexpression of barley *HvSHN1* in transgenic tobacco improved drought tolerance without compromising growth (Djemal & Khoudi, 2021). In GT31, a sugarcane variety with no tolerance to drought, *Saccharum spontaneum SsDREB1L* showed higher expression level after re-watering, indicating that *SsDREB1L* may facilitate plant recovery from drought stress (Li et al., 2021). Tobacco *ERF172* improves plant drought tolerance partly by regulating CAT-mediated H_2_O_2_ homeostasis (Zhao et al., 2020). However, in woody plants, there are little or no reports on the identification of *ERF* gene family, especially in walnut, where there is no report of *ERF* in stress response. Therefore, in this study, the walnut ERF TFs were identified and the basic bioinformation, conserved motifs, and evolutionary relationship were analysed. Meanwhile, considering that ERFs are involved in the ethylene (ETH) signalling pathway (Gu et al., 2017; Kazan, 2015; Xie et al., 2019), three stresses (PEG_6000_, ethephon, and PEG_6000_+ethephon) were used to assess the potential transcription activity of the selected *JrERF*s. The results of this study will provide profound platform for subsequent investigation of *JrERF*s response to stress.

## Materials & methods

### Plant materials and treatments

New branches were obtained from 6-year-old ‘Xiangling’ walnut (a phenotype of *J. regia* planted widely in China) and inserted into a mixture of turf peat and sand (2:1 v/v) in plastic pots and grown in a greenhouse (22 ± 2 °C, relative humidity 70 ± 5%, illumination cycle 14 h light/10 h dark) for 2 years. Considering that PEG_6000_ is a common reagent for drought simulation stress test (Abdel-Ghany et al., 2020; Ahmad et al., 2020), *ERF* is involved in ethylene signal pathway (Gu et al., 2017; Kazan, 2015; Xie et al., 2019), and ethephon (ETH) is an ethylene donor (Kim et al., 2018; Li & Tran, 2017), the 2-year-old seedlings were treated with 15% (w/v) PEG_6000_, 100 μmol/L ETH, 15% (w/v) PEG_6000_ plus 100 μmol/L ETH (PEG_6000_+ETH). Next, the leaves were collected at 0 (control), 6, 24, and 72 h, and stored at −80 °C. A fresh water-only control was conducted in parallel. RNA was isolated from all the samples. Each treatment consisted of six seedlings.

### Identification, chromosomal location, and gene structure of *JrERF*s

To identify and analyse all the members of *ERF* gene family in walnut, the *Arabidopsis*’s genome sequences of *ERF* family members were downloaded from TAIR (https://www.arabidopsis.org/) and used for homology search in walnut transcriptome, and several walnut ERF TFs were obtained. ORF Finder (https://www.ncbi.nlm.nih.gov/orffinder/) was used to find the open reading frame (ORF). Basic biological information, including amino acid number, theoretical isoelectric point (pI), and molecular weight were predicted by ExPASy (https://web.expasy.org/protparam/). The chromosomal location information of 44 *JrERF*s in the walnut genome (*Juglans microcarpa × J. regia*) were obtained from NCBI (https://www.ncbi.nlm.nih.gov/genome/?term=txid2249226[orgn) (Zhu et al., 2019). The genomic DNA sequence of *JrERF*s were obtained from NCBI (https://www.ncbi.nlm.nih.gov/) through Gene ID (Table 1), and the gene structure map of the exon-intron of *JrERF*s were determined by Gene Structure Display Server 2.0 (GSDS 2.0: http://gsds.gao-lab.org/) (Hu et al., 2015).

**Table 1:** Sequence characteristics of 44 *JrERFs*.

Gene names	Transcriptome number	GeneBank accession number	Gene ID	Chromosome site	ORF length (bp)	Number of aa	MV (kDa)	pI
JrERF01	comp22717_c0	MZ688063	LOC109004979	chr1S	1,143	380	42.47	5.02
JrERF02	comp2477_c0	MZ688064	LOC108983929	chr1D	735	244	27.29	6.04
JrERF03	comp25333_c0	MZ688065	LOC108986419	chr1D	687	228	25.83	9.89
JrERF04	comp18721_c0	MZ688066	LOC108989254	chr1D	564	187	20.98	8.76
JrERF05	comp9598_c0	MZ688067	LOC108988992	chr1D	795	264	30.03	8.97
JrERF06	comp22552_c1	MZ688068	LOC109007057	chr1D	753	250	27.86	5.42
JrERF07	comp8892_c0	MZ688069	LOC109004887	chr1D	723	240	25.84	4.98
JrERF08	comp44754_c0	MZ688070	LOC108998815	chr2S	597	198	22.37	5.12
JrERF09	comp8896_c0	MZ688071	LOC108998823	chr2S	537	178	19.68	5.29
JrERF10	comp13191_c0	MZ688072	LOC108984080	chr2S	513	170	18.34	5.26
JrERF11	comp10222_c0	MZ688073	LOC108991055	chr2S	714	237	25.61	4.80
JrERF12	comp22357_c0	MZ688074	LOC108995850	chr2S	678	225	24.82	9.04
JrERF13	comp24921_c1	MZ688075	LOC108993210	chr2S	717	238	25.85	4.99
JrERF14	comp15973_c0	MZ688076	LOC109020121	chr2S	501	166	18.25	9.78
JrERF15	comp16155_c0	MZ688077	LOC108980339	chr2D	1,185	394	42.83	6.20
JrERF16	comp23695_c0	MZ688078	LOC109013239	chr2D	1,161	386	42.8	5.77
JrERF17	comp12993_c0	MZ688079	LOC108986643	chr2D	678	225	24.31	9.27
JrERF18	comp27438_c0	MZ688080	LOC108993692	chr2D	522	173	18.95	6.97
JrERF19	comp9852_c0	MZ688081	LOC109020467	chr2D	615	204	22.52	4.94
JrERF20	comp18782_c0	MZ688082	LOC108995608	chr2D	636	211	23.61	6.98
JrERF21	comp55856_c0	MZ688083	LOC108995945	chr2D	510	169	18.85	9.48
JrERF22	comp22253_c0	MZ688084	LOC108992157	chr3S	675	224	24.53	5.47
JrERF23	comp26055_c0	MZ688085	LOC108981535	chr3S	1,158	385	42.45	7.14
JrERF24	comp10353_c0	MZ688086	LOC108982941	chr3S	762	253	28.61	5.38
JrERF25	comp26003_c0	MZ688087	LOC108997399	chr3D	681	226	24.95	6.10
JrERF26	comp25379_c1	MZ688088	LOC108984027	chr3D	1,107	368	40.07	6.01
JrERF27	comp18282_c0	MZ688089	LOC109002490	chr4S	708	235	26.17	7.6
JrERF28	comp29196_c0	MZ688090	LOC108994146	chr4D	963	320	35.96	5.08
JrERF29	comp40170_c0	MZ688091	LOC108994146	chr4D	1,002	333	37.79	5.79
JrERF30	comp10012_c0	MZ688092	LOC108992195	chr4D	615	204	22.86	8.75
JrERF31	comp14427_c0	MZ688093	LOC108984175	chr5D	489	162	17.47	9.83
JrERF32	comp9632_c0	MZ688094	LOC108984188	chr5D	732	243	26.35	9.71
JrERF33	comp26703_c0	MZ688095	LOC109010126	chr6S	939	312	34.25	6.75
JrERF34	comp23499_c0	MZ688096	LOC108997304	chr6S	981	326	37.09	5.09
JrERF35	comp53300_c0	MZ688097	LOC108990846	chr6S	702	233	25.21	5.46
JrERF36	comp20406_c0	MZ688098	LOC108994010	chr6S	714	237	26.08	8.80
JrERF37	comp21129_c0	MZ688099	LOC109005134	chr6D	870	289	31.16	8.18
JrERF38	comp18221_c1	MZ688100	LOC109005151	chr6D	990	329	36.52	7.10
JrERF39	comp23713_c0	MZ688101	LOC108989912	chr6D	762	253	27.99	6.96
JrERF40	comp29151_c0	MZ688102	LOC109013315	chr6D	654	217	23.51	7.02
JrERF41	comp22664_c0	MZ688103	LOC108981529	chr8S	1,239	412	43.71	7.04
JrERF42	comp28472_c0	MZ688104	LOC108990403	chr8S	450	149	16.81	8.92
JrERF43	comp17398_c0	MZ688105	LOC109021567	chr8S	999	332	36.36	6.27
JrERF44	comp21821_c0	MZ688106	LOC108994552	chr8D	1,011	336	37.48	5.36

**Note:**

Amino acid, aa; Molecular weight, MV; Theoretical isoelectric point, pI.

### Multiple sequence alignment, conserved domain, and phylogenetic analysis of JrERFs

CD-Search (https://www.ncbi.nlm.nih.gov/Structure/cdd/wrpsb.cgi), Pfam (http://pfam.xfam.org/), and SMART (http://smart.embl-heidelberg.de/) were used to analyse the conserved domains of JrERFs. Multi-sequence alignment was applied using ClustalX2.1. MEME online tools (http://alternate.meme-suite.org/) were adopted to uncover the conservative motifs. The setting parameters were as follows: the number of motifs was 20, any number repetition was allowed, motif width was from 6 to 50. We downloaded 63 *A. thaliana* ERF proteins from TAIR, 37 *P. trichocarpa* ERFs from Joint Genome Institute *P. trichocarpa* version 1.1 database (https://mycocosm.jgi.doe.gov/Poptr1_1/Poptr1_1.home.html). The ERF proteins from *A. thaliana*, *P. trichocarpa*, and *J. regia* were used to construct a neighbour-joining tree for evolutionary analysis with a bootstrap replicate value of 1,000 using MEGA7. The phylogenetic tree was modified using iTOL (https://itol.embl.de/) (Letunic & Bork, 2021).

### Expression analysis of *JrERF*s by real-time quantitative polymerase chain reaction (qRT-PCR) (cetyltrimethylammonium ammonium bromide)

The total RNA of each sample was isolated using the CTAB method (Yang et al., 2018) and digested with DNase (Takara, Dalian, China). Next, 0.5 μg RNA of each sample was reverse transcribed into cDNA using PrimeScript™ RT reagent Kit (CWBIO, Beijing, China). The cDNA was diluted 10-fold by sterile water and used as the template of qRT-PCR. The reaction mixture (20 μL) contained 10 μL of SYBR Green Real-time PCR Master Mix (CWBIO, Beijing, China), 0.5 μM of each forward and reverse primer, and 2 μL cDNA template (equivalent to 100 ng of total RNA). qRT-PCR was performed in StepOne™ Real-Time PCR System produced by Applied Biosystems. The amplification was achieved according to the following parameters: 94 °C for 30 s, followed by 44 cycles at 94 °C for 12 s, 60 °C for 30 s, 72 °C for 40 s, and at 81 °C for 1 s. The internal reference gene is walnut *18S rRNA* (HE574850) (Xu et al., 2012), and the primers are shown in Table S1. The relative expression levels were calculated based on the threshold cycle using the 2^−ΔΔCT^ method (Livak & Schmittgen, 2001).

## Results

### Sequence characteristics and chromosomal locations of *JrERF*s

In summary, a total of 44 *JrERF*s were identified from walnut transcriptome. The ORFs of *JrERF*s were between 450 bp (*JrERF42*) and 1,239 bp (*JrERF41*), consisting 149–412 amino acids. The molecular weight of the proteins ranged from 16.81 kDa (*JrERF42*) to 43.71 kDa (*JrERF41*), and the pI ranged from 4.80 (*JrERF11*) to 9.89 (*JrERF03*) (Table 1).

**Figure 1:**
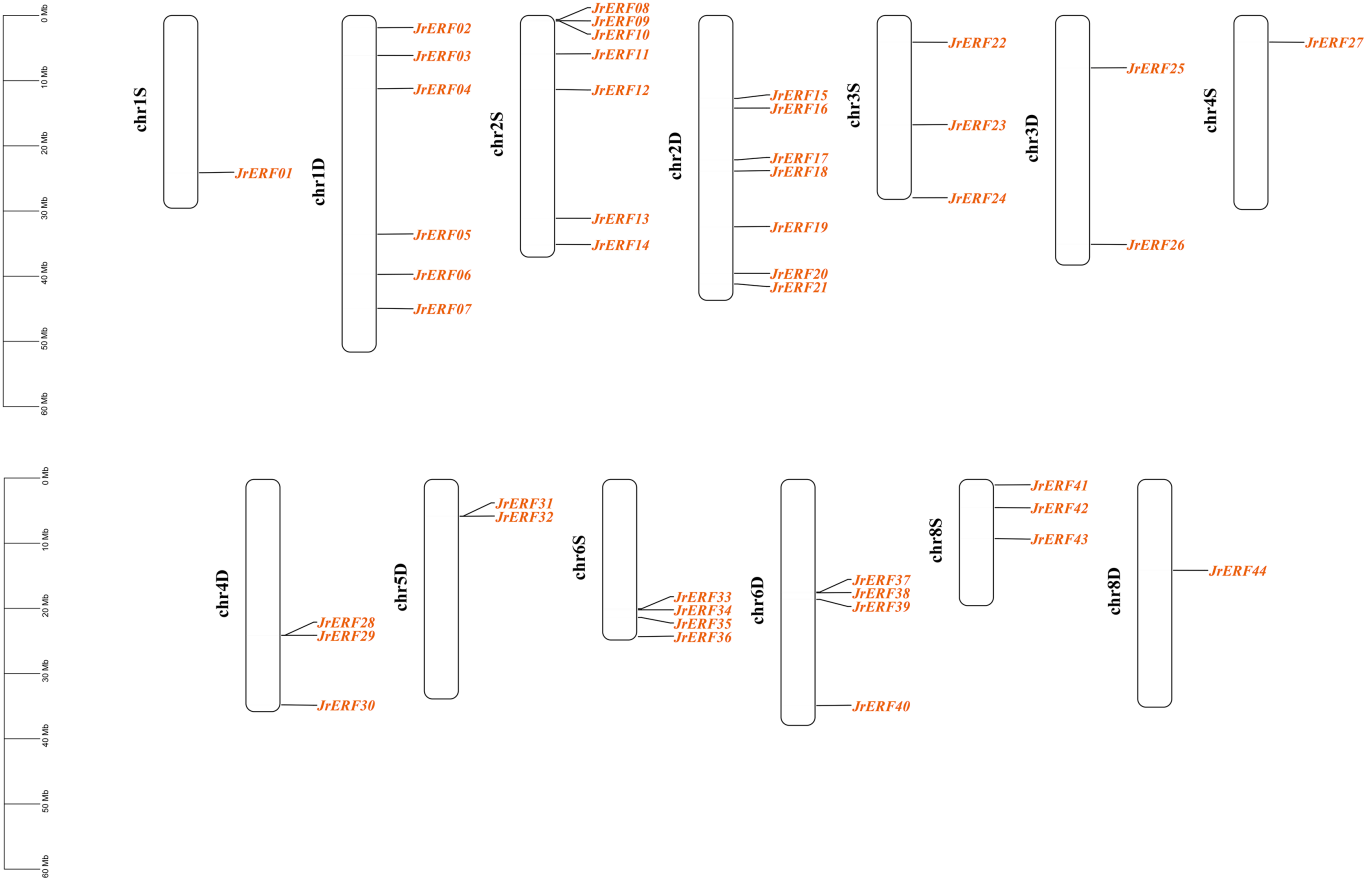
Distribution of the *JrERFs* on chromosomes of *J. regia*. The chromosome number is shown on the left side of each chromosome, D: Dominant; S: Subdominant.

These 44 *JrERF*s were unevenly located on 13 chromosomes (chr1S, chr1D, chr2S, chr2D, chr3S, chr3D, chr4S, chr4D, chr5D, chr6S, chr6D, chr8S, chr8D) of *J. regia*. The chromosomes 1D, 2S, and 2D covered the most number of *JrERF*s (6, 7, and 7 genes, respectively), while the chromosomes 1S, 4S and 8D included the least number of *JrERFs* (1 gene, accordingly). The chromosomes 5S, 7S, and 7D contained no *JrERF*s (Fig. 1).

**Figure 2:**
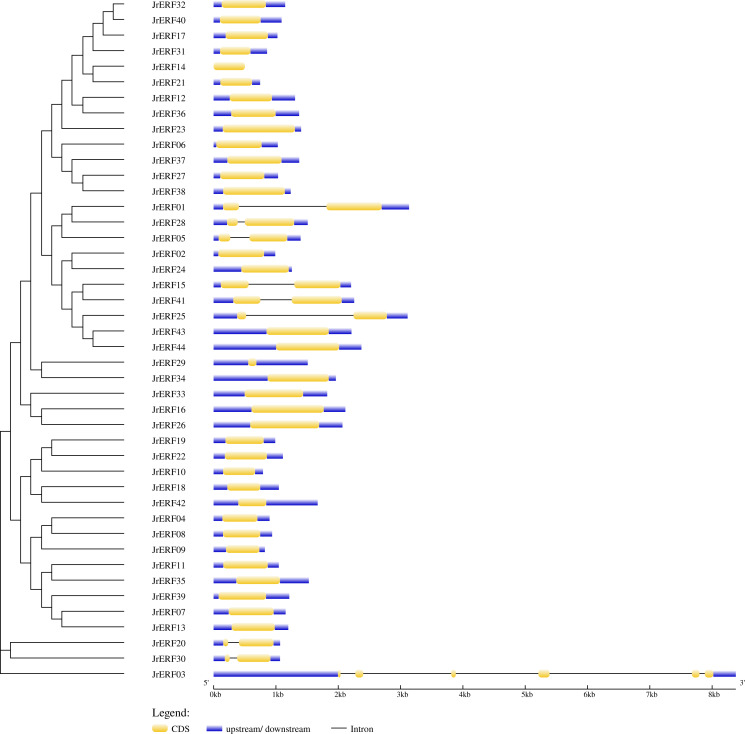
Gene structure map of *JrERFs*. The vertical phylogenetic tree and gene structure of *JrERFs* was constructed by GSDS online software. Yellow boxes indicate exons; blue boxes indicate upstream or downstream; black lines indicate introns.

The intron and exon structure of *JrERF*s were analysed by comparing the genomic DNA sequence of *JrERF*s. The results showed that the gene structures of nine *JrERF*s (*JrERF01*, *JrERF03*, *JrERF05*, *JrERF15*, *JrERF20*, *JrERF25*, *JrERF28*, *JrERF30*, *JrERF41*) were destroyed by introns, and the other 35 *JrERF*s contained no intron and their structures are relatively stable (Fig. 2).

### The conserved domains of JrERFs

**Figure 3:**
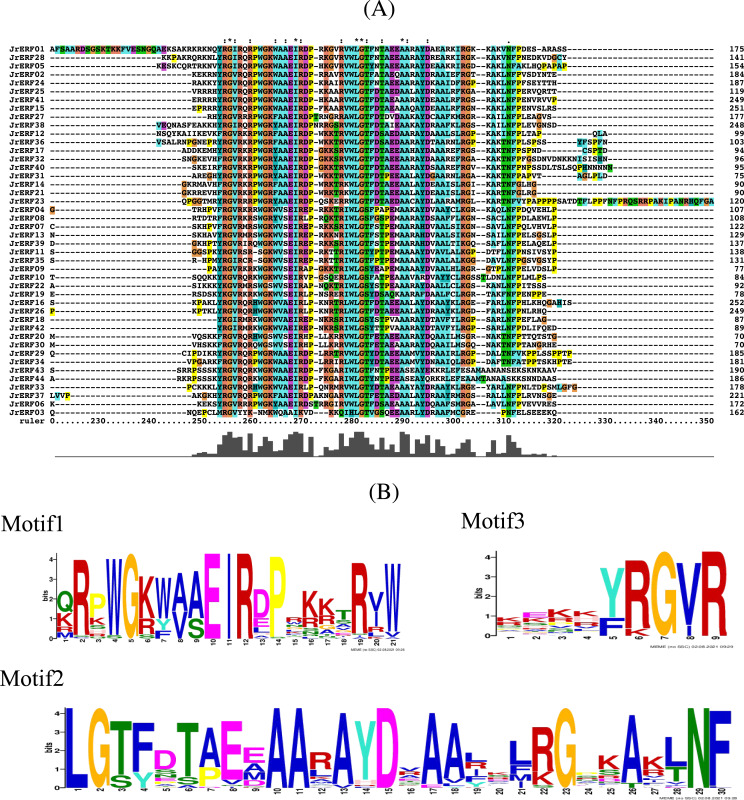
Multiple sequence alignment of the JrERFs proteins. (A) Multiple sequence alignment of the AP2s of JrERF proteins. ‘:’ means the mutation at that position is a conservative mutation; ‘.’ means a semi-conservative mutation; ‘*’ indicates that the sequence is consistent at that site. (B) The seqlogos of motif1, motif2 and motif3.

Conserved domain analysis showed that there was a common AP2 conserved domain with different degrees of insertion or deletion in the JrERF proteins (Fig. 3A; Fig. S1). MEME and Tbtools (Chen et al., 2020) were used to construct the conserved domain sequence and sequence logo, and the result suggested that most JrERFs contain conserved motifs. Three motifs (motif1, motif2, and motif3) existed in most of the sequences. We found that motif1–3 may be part of the AP2 domain; sequence similarity was 100% at sites 5, 10, 11, and 12 in motif1, sites 1, 2, 10, 11, 15, 23, and 29 in motif2, and sites 7 and 9 in motif3 (Fig. 3B).

### The conservative motif of JrERFs

**Figure 4:**
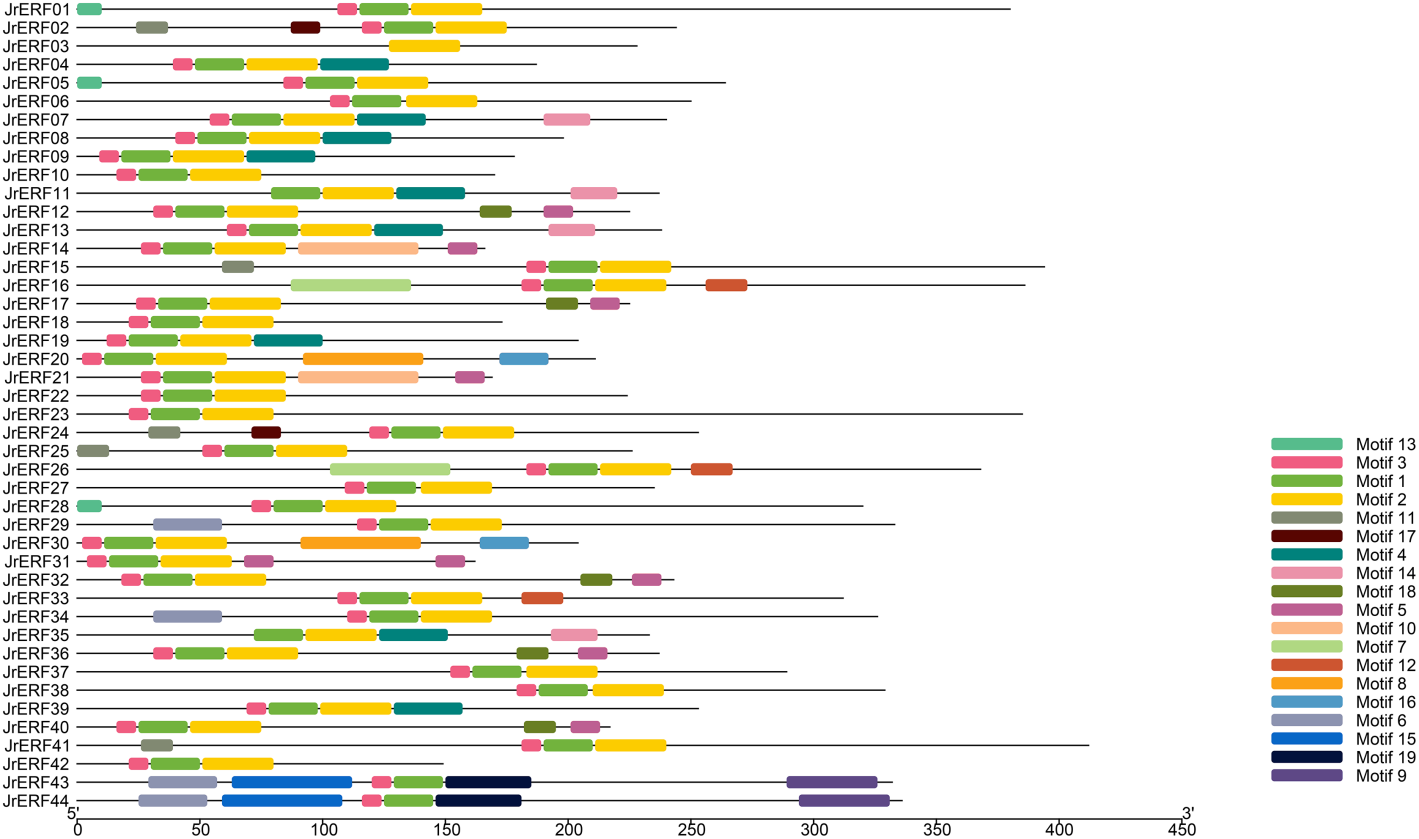
Distribution of the conserved motifs in JrERF proteins. The different colours represent different motifs (motif1—motif19).

We used MEME to analyse the motifs in the 44 JrERFs and downloaded the basic information (width and best possible match sequence) (Table 2). The results showed that each motif contained 9–50 amino acids, and each sequence contains 1–6 motifs. Among 44 amino acid sequences, JrERF43 and JrERF44 had six conserved motifs (motif6, motif15, motif3, motif1, motif19, motif9), while JrERF03 had only one (motif2). JrERF12, 17, 32, 36, and 40 had five (motif3, motif1, motif2, motif18, motif5). In addition, the most frequent motifs of JrERFs are motif1 (QRPWGKWAAEIRDPRKKTRVW), motif2 (LGTFDTAEEAARAYDRAALKLRGPKAKLNF), and motif3 (KEKKYRGVR). They represented the AP2 domain and are widely present in the 44 JrERFs (Fig. 4).

**Table 2:** Motif sequences identified by MEME tool.

motif	Width	Best possible match
motif1	21	QRPWGKWAAEIRDPRKKTRVW
motif2	30	LGTFDTAEEAARAYDRAALKLRGPKAKLNF
motif3	9	KEKKYRGVR
motif4	29	PELVESLPRPASSSPRDIQAAAAKAAAMK
motif5	13	RRPLPFDLNLPPP
motif6	29	KMRVVRIIVSDPYATDSSSSEDDSEKCVK
motif7	50	TPMFSEGFSSQNQMGFEQPGPJGLNQLTPSQILQIQAQIQLQKQNQQRQQ
motif8	50	KLRKCCKDPYPSLTCLRLDAENSHIGVWQKRAGQRSDSNWIMRIPLGKKN
motif9	38	FFDDFEVCGTEDGGGNELPDWDFADICDDFGWMNEPLN
motif10	50	LVITPPVLSGGAGACELFGWRPKPECFSGAGNPPPVRSEYKGYKMENVDV
motif11	14	MSIMVSALTHVVSG
motif12	18	DYKPLHSSVDAKLQAICQ
motif13	11	MCGGAIISDFI
motif14	20	MPNLLVDMAEGMLVSPPRIN
motif15	50	KRLVREIHJPLVKQPPPKLLQSESSCQDSNNGGRTPKVIEAEKKRVLAKT
motif16	21	MDEEERIALQMIEELLNWNCP
motif17	13	CPVCNIDGCLGCN
motif18	14	GCHSDSDSSSVVDD
motif19	36	LGTFNTPEEASEAYZKKRLEFEAAMAANANSEKSKN

### The evolutionary relationship of JrERFs

**Figure 5:**
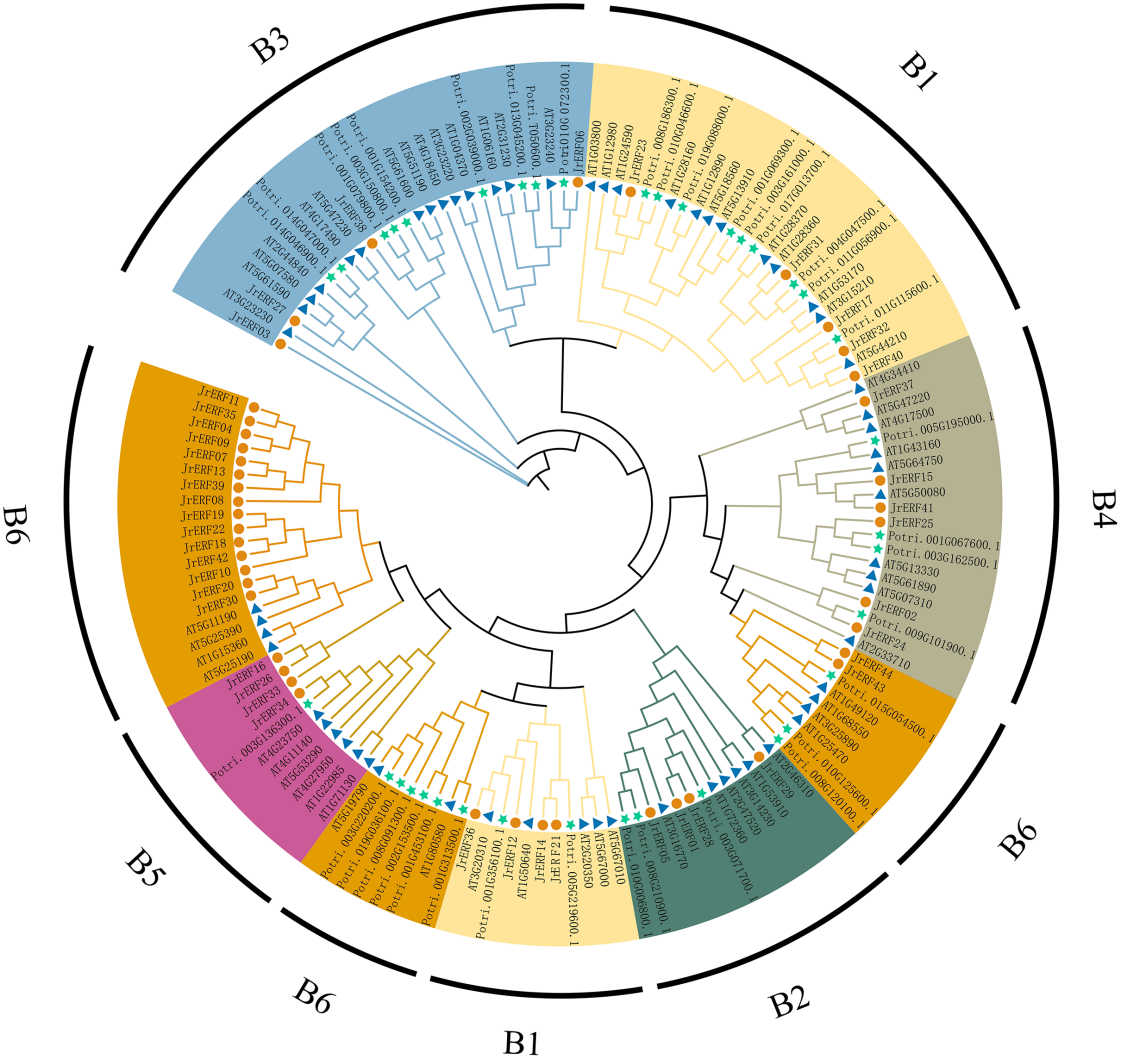
Phylogenetic relationship of ERF proteins from *J. regia*, *A. thaliana* *and P. trichocarpa*. B1–B6 means six groups of *JrERFs*, respectively, which are displayed in different colours. A total of 63 *Arabidopsis* ERFs are represented by blue triangles, 44 walnut ERFs are represented by vermilion circles, 37 *P. trichocarpa* ERFs are represented by green five-pointed stars.

To analyse the evolutionary relationships of JrERFs, 44 JrERFs, 63 *A. thaliana* ERFs, and 37 *P. trichocarpa* ERFs were aligned for the construction of the phylogenetic tree. The results showed that JrERFs can be classified into six groups (B1–B6). Group B6 contained the highest number of JrERF proteins (17 members); groups B2, B3, and B5 had only four members they respectively include JrERF01, JrERF05, JrERF28, JrERF29; JrERF03, JrERF06, JrERF27, JrERF38; JrERF16, JrERF26, JrERF33, JrERF34; groups B4 contained six members (JrERF02, JrERF15, JrERF24, JrERF25, JrERF37, JrERF41); and the remaining nine JrERF proteins were in group B1 (Fig. 5).

### Expression of *JrERF*s in response to drought stress

**Figure 6:**
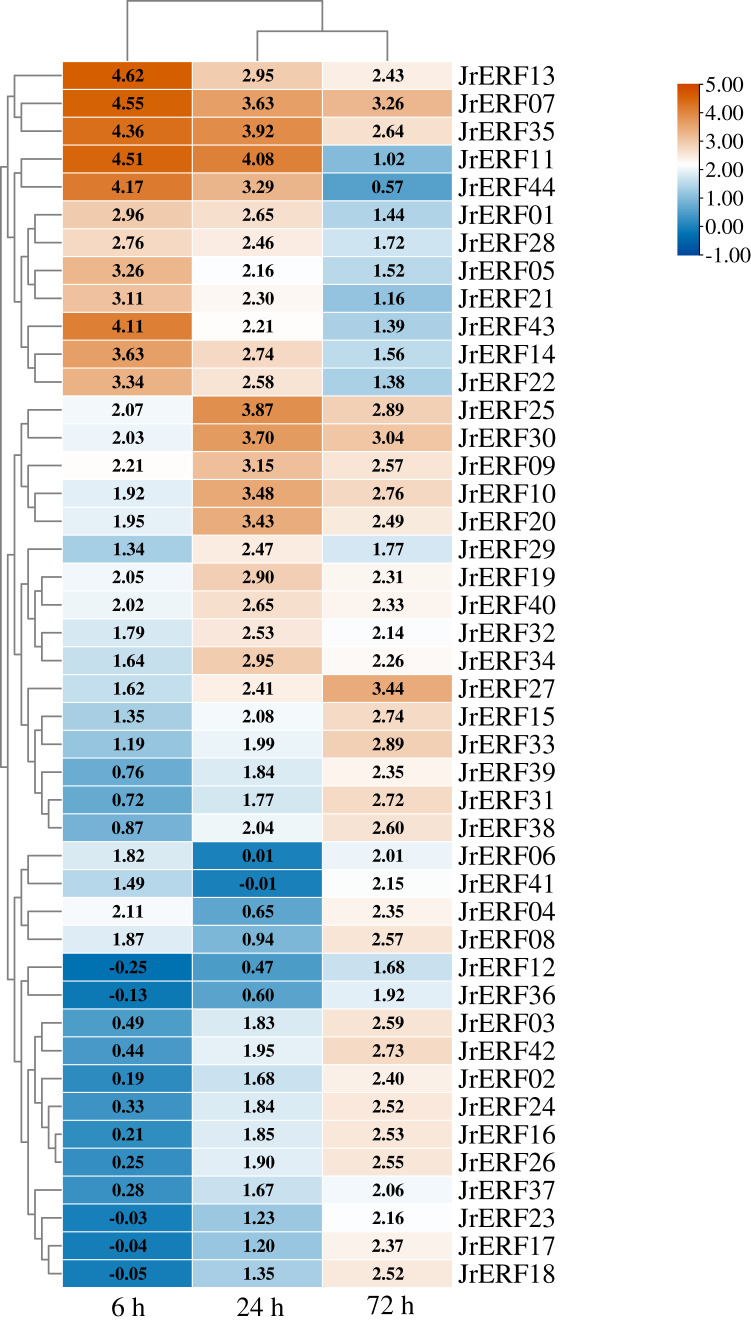
The relative expression of the 44 *JrERFs* under PEG_6000_ stress. The expression is relative to the expression of the internal reference gene and at 0 h.

**Figure 7:**
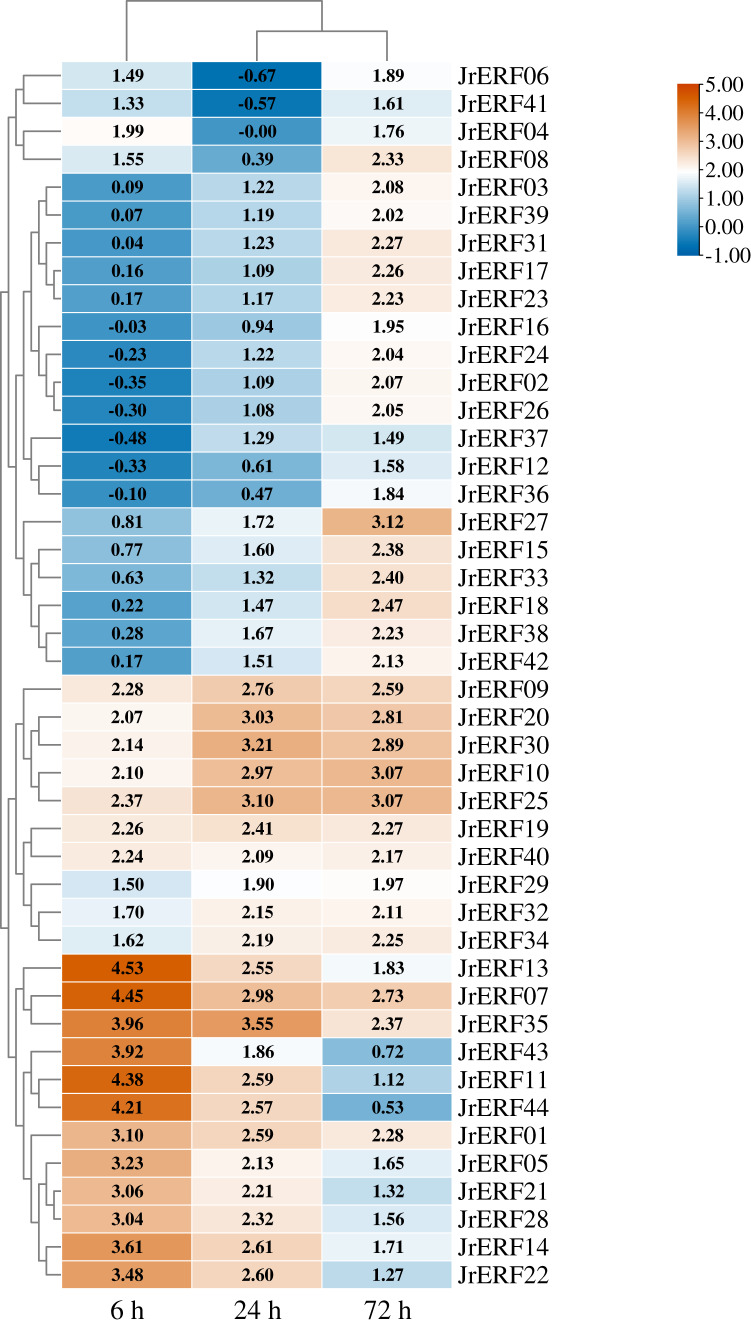
The relative expression of the 44 *JrERFs* under ethephon (ETH) stress. The expression is relative to the expression of the internal reference gene and at 0 h.

**Figure 8:**
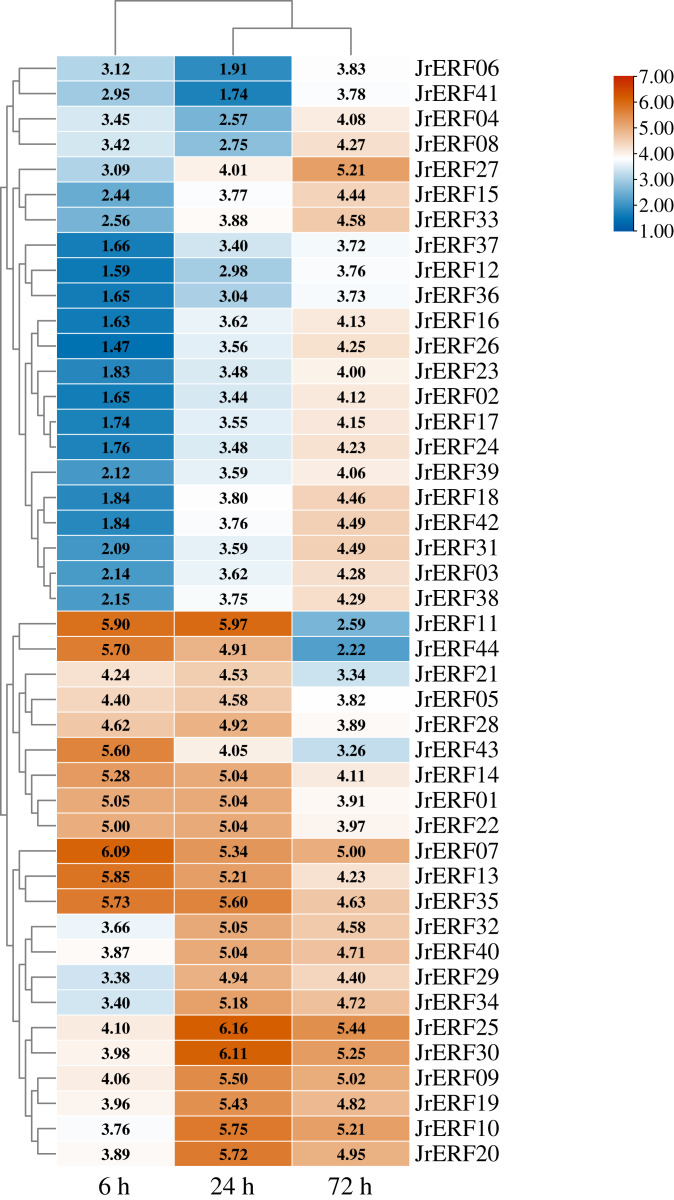
The relative expression of the 44 *JrERFs* under PEG_6000_+ETH stress. The expression is relative to the expression of the internal reference gene and at 0 h.

To explore the potential function of *JrERF*s in response to drought stress, the expression of all the *JrERF*s were analysed under PEG_6000_, ETH, and PEG_6000_+ETH treatments (Figs. 6–8).

### Under PEG_6000_ stress

The expression levels of 44 *JrERF*s under PEG_6000_ stress showed four different trends. (i) The expression of 18 *JrERF*s (*JrERF02*, *JrERF03*, *JrERF12*, *JrERF15*, *JrERF16*, *JrERF17*, *JrERF18*, *JrERF23*, *JrERF24*, *JrERF26*, *JrERF27*, *JrERF31*, *JrERF33*, *JrERF36*, *JrERF37*, *JrERF38*, *JrERF39*, *JrERF42*) showed a continuous increasing trend at 6–72 h; the expression level of *JrERF27* at 72 h was 2.05-fold higher than that of *JrERF12*. (ii) The expression levels of 12 *JrERF*s (*JrERF01*, *JrERF05*, *JrERF07*, *JrERF11*, *JrERF13*, *JrERF14*, *JrERF21*, *JrERF22*, *JrERF28*, *JrERF35*, *JrERF43*, *JrERF44*) showed a continuous decreasing trend at 6–72 h; *JrERF11* had the highest expression at 6 h, which was 1.21-fold higher than the average expression level of the other genes in the group. (iii) The expression levels of 10 *JrERF*s (*JrERF09*, *JrERF10*, *JrERF19*, *JrERF20*, *JrERF25*, *JrERF29*, *JrERF30*, *JrERF32*, *JrERF40*, *JrERF34*) increased at 6–24 h and decreased at 24–72 h. The average expression value of this group was 3.11 at 24 h. (iv) The relative expression levels of four *JrERF*s (*JrERF04*, *JrERF06*, *JrERF08*, *JrERF41*) decreased at 6–24 h and increased at 24–72 h. *JrERF04* showed the highest expression at 6 h, which was 1.41-fold higher than that of *JrERF41* (Fig. 6).

### Under ETH stress

Four different expression trends were observed when the 44 *JrERF*s were subjected to ETH treatment. (i) The expression levels of 21 *JrERF*s (*JrERF02*, *JrERF03*, *JrERF10*, *JrERF12*, *JrERF15*, *JrERF16*, *JrERF17*, *JrERF18*, *JrERF23*, *JrERF24*, *JrERF26*, *JrERF27*, *JrERF29*, *JrERF31*, *JrERF33*, *JrERF34*, *JrERF36*, *JrERF37*, *JrERF38*, *JrERF39*, *JrERF42*) showed a continuous increasing trend at 6–72 h, and the expression level of *JrERF27* at 72 h was 2.09-fold of *JrERF37. JrERF10* and *JrERF27* maintained high expression after 72 h. (ii) The relative expression of 12 *JrERF*s (*JrERF01*, *JrERF05*, *JrERF07*, *JrERF11*, *JrERF13*, *JrERF14*, *JrERF21*, *JrERF22*, *JrERF27*, *JrERF35*, *JrERF43*, *JrERF44*) showed a continuous declining trend at 6–72 h, and the average expression level was 3.75 at 6 h. (iii) The expression levels of six *JrERFs* (*JrERF09*, *JrERF19*, *JrERF20*, *JrERF25*, *JrERF30*, *JrERF32*) increased at 6–24 h and decreased at 24–72 h. The expression level of *JrERF20* at 6 h was 1.22-fold higher than that of *JrERF32*. (iv) The relative expression of five *JrERF*s (*JrERF04*, *JrERF06*, *JrERF08*, *JrERF40*, *JrERF41*) decreased at 6–24 h and increased at 24–72 h. At 6 h, *JrERF40* showed the highest expression, which was 1.41-fold higher than the average expression level of the other genes in the group (Fig. 7).

### Under PEG_6000_+ETH stress

*JrERF*s displayed four different expression trends under PEG_6000_+ETH stress. (i) The expression levels of 18 *JrERF*s (*JrERF02*, *JrERF03*, *JrERF12*, *JrERF15*, *JrERF16*, *JrERF17*, *JrERF18*, *JrERF23*, *JrERF24*, *JrERF26*, *JrERF27*, *JrERF31*, *JrERF33*, *JrERF36*, *JrERF37*, *JrERF38*, *JrERF39*, *JrERF42*) showed a continuous increase at 6–72 h; *JrERF27* and *JrERF33* maintained high expression after 72 h, and the expression level of *JrERF27* at 72 h was 1.40-fold higher than that of *JrERF37*. (ii) The relative expression of seven *JrERF*s (*JrERF01*, *JrERF07*, *JrERF13*, *JrERF14*, *JrERF35*, *JrERF43*, *JrERF44*) showed a continuous decreasing trend at 6–72 h, and the average expression value was 5.61 at 6 h in this group. (iii) The expression levels of 15 *JrERF*s (*JrERF05*, *JrERF09*, *JrERF10*, *JrERF11*, *JrERF19*, *JrERF20*, *JrERF21*, *JrERF22*, *JrERF25*, *JrERF28*, *JrERF29*, *JrERF30*, *JrERF32*, *JrERF34*, *JrERF40*) increased at 6–24 h and decreased within 24–72 h, and the average expression level was 5.33 at 24 h. (iv) The expression levels of four *JrERF*s (*JrERF04*, *JrERF06*, *JrERF08*, *JrERF41*) showed a decreasing trend at 6–24 h and increasing trend at 24–72 h, and their expression levels were higher than 3.50 at 72 h (Fig. 8).

These results showed that the expression of 44 *JrERF*s in the presence of ETH was higher than that observed with PEG_6000_ stress alone. It is worth mentioning that the expression levels of seven *JrERF*s (*JrERF01*, *JrERF07*, *JrERF13*, *JrERF14*, *JrERF35*, *JrERF43*, *JrERF44*) exceeded 5.00 at 6 h under the PEG_6000_+ETH stress, with *JrERF07* showing the highest expression (6.09). These findings indicate that in the presence of ETH, the relative expression of *JrERF*s to drought stress is enhanced, and some *JrERF*s have potential drought resistance function.

## Discussion

Walnut is an important economic species, and like other plants, its growth and development are restricted by adverse environmental conditions (Zhang et al., 2020b). There are little or no reports on ERF, a big subfamily of AP2/ERF playing important roles in stress response, in walnut tree. To increase the yield and quality of walnuts as well as ensure farmers’ economic income and stable development of walnut industry under adverse conditions, it is necessary to elucidate the molecular mechanism associated with adversity adaptation in walnut. Therefore, in this study, a total of 44 *JrERF*s that may have potential functions in drought stress response were obtained from walnut transcriptome and divided into six groups (Fig. 5). This classification was consistent with previous evolutionary analyses of *Apium graveolens* (Li et al., 2019a), *Zay mays* (Hao et al., 2020), and *Dimocarpus longan* (Zhang et al., 2020a). The various characteristics including ORF length, pI, amino acid number, and molecular weight of the walnut ERF family had a large span (Table 1). The phenomenon is similar to the sequence characteristics of *Arabidopsis* and *P. trichocarpa* (Zhuang et al., 2008), indicating that the ERF family of walnut has a certain similarity in sequence characteristics with other species.

Multiple alignment result showed that the 44 JrERFs are highly conserved with AP2 domain (Fig. 3A; Fig. S1), and this was consistent with the conserved region of AtERFs (Fig. S2). This result further confirmed that ERF TFs have highly conserved structures in procession of species evolutionary (Nakano et al., 2006). Gene structure analysis showed that 79.55 % of the *JrERF*s had no intron, implying that most *JrERF*s have relatively stable gene structure (Fig. 2), which was similar to that observed with *AP2/ERF* genes from tartary buckwheat (*Fagopyum tataricum*) (Liu et al., 2019). Most JrERFs contained motif1, motif2, and motif3, which were related to AP2 domain (Fig. 3B). Motif1–motif19 were distributed in the 44 JrERFs at different degrees (Fig. 4), which was also similar to what was observed with ERF TFs from *F. tataricum* and *Medicago sativa* (Jin et al., 2019; Liu et al., 2019). Because different motifs and the number of motifs are related to functions (Tripathi et al., 2020), our results suggest that *JrERF*s may play different roles in plant stress response or other biological functions.

Considering that drought has a great impact on the walnut industry (Knipfer et al., 2018), the responses of the identified 44 *JrERFs* to PEG_6000_ were analysed in order to uncover the functions of walnut AP2/ERF family in response to drought stress. The results showed that most of the *JrERF*s were induced by PEG_6000_ (Fig. 6). The expression of genes in response to different stresses can often effectively predict their potential functions. For example, it has been reported that the expression levels of various AP2/ERF TFs (*Bra-ERF036*, *Bra-ERF069a*, and *Bra-ERF104a*) from *Brassica oleracea* are rapidly up-regulated by drought stress, which confirms their important roles in tolerance to abiotic stress (Li et al., 2017). *IbRAP2-12*, a member of sweet potato ERF family, was rapidly enhanced by drought stress and played a crucial role in enhancing plant tolerance to drought stress (Li et al., 2019b). The expression of *JrWRKY2* and *JrWRKY7* from walnut was enhanced by PEG_6000_ treatment, and both were further confirmed to be positive TFs in response to drought stress (Yang et al., 2017). According to these findings, we speculate that *JrERF*s can respond to drought stress to varying degrees and some members may play positive roles in the regulation of drought tolerance.

Ethylene, a plant growth regulator, participates in multiple plant stress response, such as drought, low temperature, salinity, and mechanical damage (Bleecker & Kende, 2000; Johnson & Ecker, 1998; Mizoi, Shinozaki & Yamaguchi-Shinozaki, 2012). In drought response, ethylene plays a positive role in enhancing drought resistance (Wan et al., 2011). ERFs are associated with ethylene response, and most *ERF*s can be regulated by ethylene (Müller & Munné-Bosch, 2015; Xie et al., 2019). For instance, the *OsDERF1* from rice can be activated by ethylene to improve tolerance to drought stress (Wan et al., 2011). Therefore, in order to verify that the response of *JrERF*s under drought is related to the ethylene signal pathway, walnuts were treated with ETH and PEG_6000_+ETH, and the expression levels of *JrERF*s were analysed. Furthermore, the expression levels of *JrERF*s under the two treatments were compared. The results showed that most *JrERF*s can be up-regulated by ETH (Fig. 7), suggesting that ETH has regulatory effect on the expression of *JrERF*s. Moreover, under PEG_6000_+ETH, the expression levels of 44 *JrERF*s were obviously higher than those observed under PEG_6000_ alone (Fig. 8). These results are similar to other reports. For instance, the expression level of *ZmEREB180* was significantly improved with increase in the level of ethylene, which benefited the waterlogging tolerance of plant, indicating that the drought response role of *ZmEREB180* was mediated by ethylene (Yu et al., 2019). *Arabidopsis RAP2.2* regulated the ability of plants to resist hypoxia through ethylene-controlled signal transduction pathways (Hinz et al., 2010). In soybean, ethylene treatment significantly enhanced the expression of *GmERF3*, and the overexpression of *GmERF3* in tobacco improved the salt and drought tolerance of transgenic lines (Zhang et al., 2009). Therefore, we can conclude that the ethylene signalling pathway is involved in the response of *JrERF*s to drought stress.

In addition, among the 44 *JrERF*s, *JrERF11* showed the highest expression. The expression of *JrERF11* reached the peak at 24 h (5.97) under PEG_6000_+ETH stress, which was 1.41-fold higher than that of other *JrERFs* at 24 h. *JrERF11* contains no intron and is evolutionarily close to *PbERF027* from *Pyrus brestschneideri* (Fig. S3). *PbERF027* is involved in the regulation of genes related to plant hormones (Pei et al., 2016). Moreover, the expression of *NnERF026*, another homologue from *Nelumbo nucifera* (Fig. S3), could be induced by drought stress and may function positively in drought stress (Cao et al., 2021). Based on these findings, it can be speculated that *JrERF11* is a potential useful gene for drought tolerance and its function should be further investigated.

## Conclusions

In this study, a total of 44 *JrERF*s were identified from *J. regia* and their basic biological information, chromosome locations, gene structure, conserved motifs, phylogenetic relationship were analysed. We found that the *JrERF*s were highly conserved and belonged to six groups. More than 40% of the *JrERF*s can be induced by drought stress in the presence of ETH, implying that *JrERF*s can respond to drought stress through the ethylene signalling pathway. *JrERF11* is the most prominently expressed gene and worthy of further study. The result of our study can provide solid foundation for further investigation of *JrERF*s under multiple abiotic stresses and for exploring the molecular mechanism underlying abiotic stress responses in walnut and other woody plants.

## Supplemental Information

10.7717/peerj.12429/supp-1 Supplemental Information 1 The AP2 domain of 44 JrERFs proteins.

10.7717/peerj.12429/supp-2 Supplemental Information 2 Multiple sequence alignment of the AP2s of 44 JrERFs proteins and 63 *A. thaliana* ERF proteins.

10.7717/peerj.12429/supp-3 Supplemental Information 3 Phylogenetic tree analysis of JrERF11 protein and the homologous from other species. Tc: *Theobroma cacao*; Hu: *Herrania umbratica*; Dz: *Durio zibethinus*; Pa: *populus alba*; Jc: *Jatropha curcas*; Pb: *Pyrus brestschneideri;* Zj*: Ziziphus jujuba;* Jr*: Juglans regia;* Cc: *Citrus clementina*; Nn: *Nelumbo nucifera*.

10.7717/peerj.12429/supp-4 Supplemental Information 4 The primers for qRT-PCR analysis under different stresses and tissues.

10.7717/peerj.12429/supp-5 Supplemental Information 5 qRT-PCR raw data.

10.7717/peerj.12429/supp-6 Supplemental Information 6 Full information of the *JrERFs* from transcriptome annotation including Gene Ontology.

## Additional Information and Declarations

### Data Availability

The following information was supplied regarding data availability: The GenBank accession numbers of 44 *JrERF*s are available in Table 1. The full information of the *JrERF*s from transcriptome annotation including Gene Ontology and qRT-PCR raw data are available in the Supplemental Files.
