# Supplementary figures and images for: Genome-wide identification and expression analysis of ethylene responsive factor family transcription factors in Juglans regia
Source: PeerJ. 2021 Nov 19;9:e12429. doi: 10.7717/peerj.12429 (PMC8607932; doi:10.7717/peerj.12429)

AP2

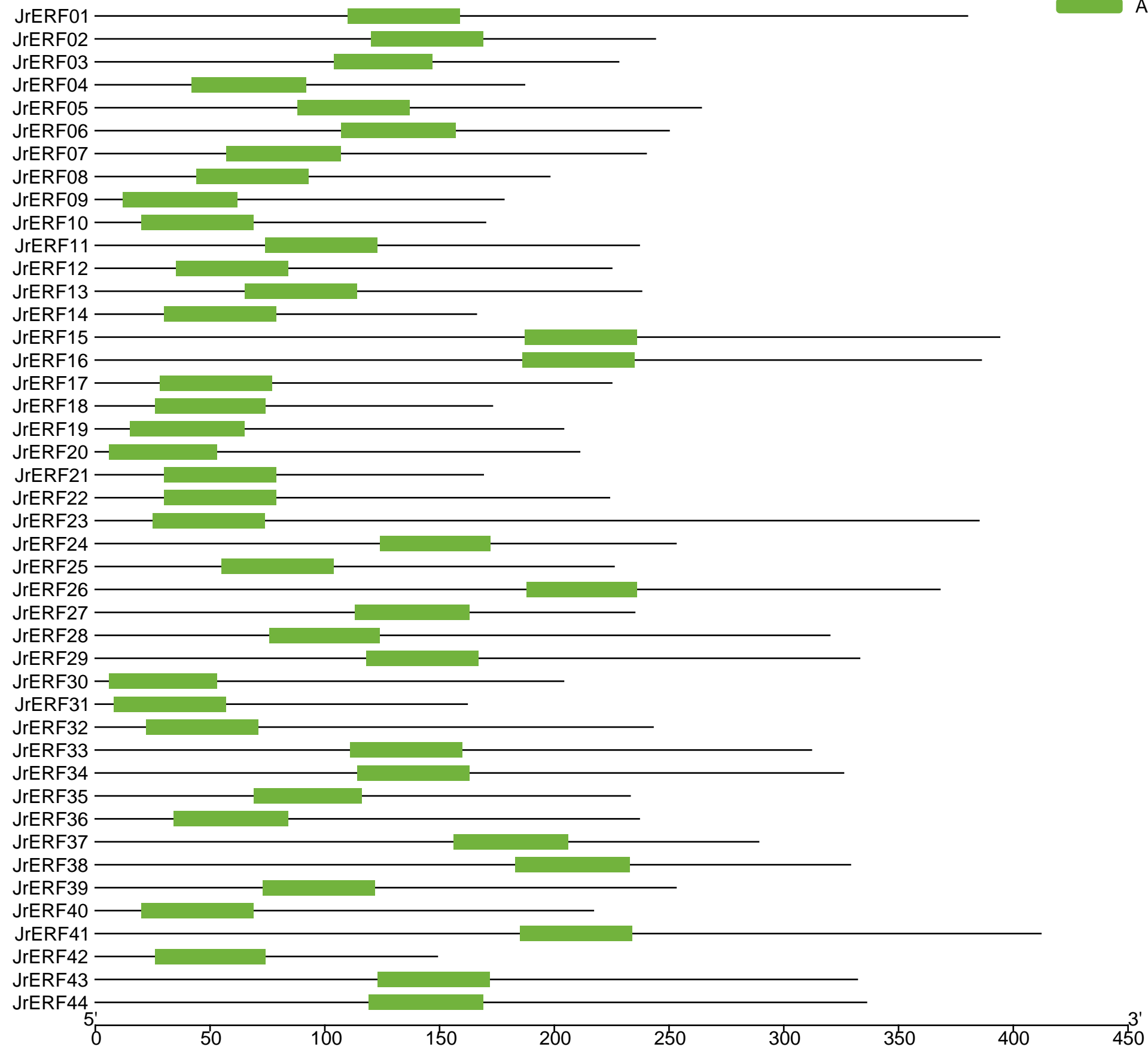

Supplement: Supplemental Information 1 [file peerj-09-12429-s001.pdf]

[illegible]



[illegible]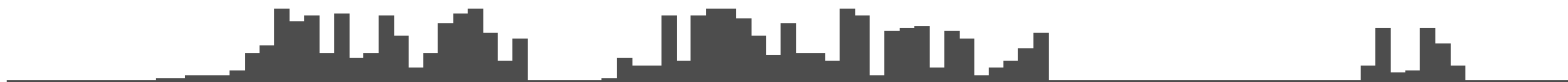

Supplement: Supplemental Information 2 [file peerj-09-12429-s002.pdf]

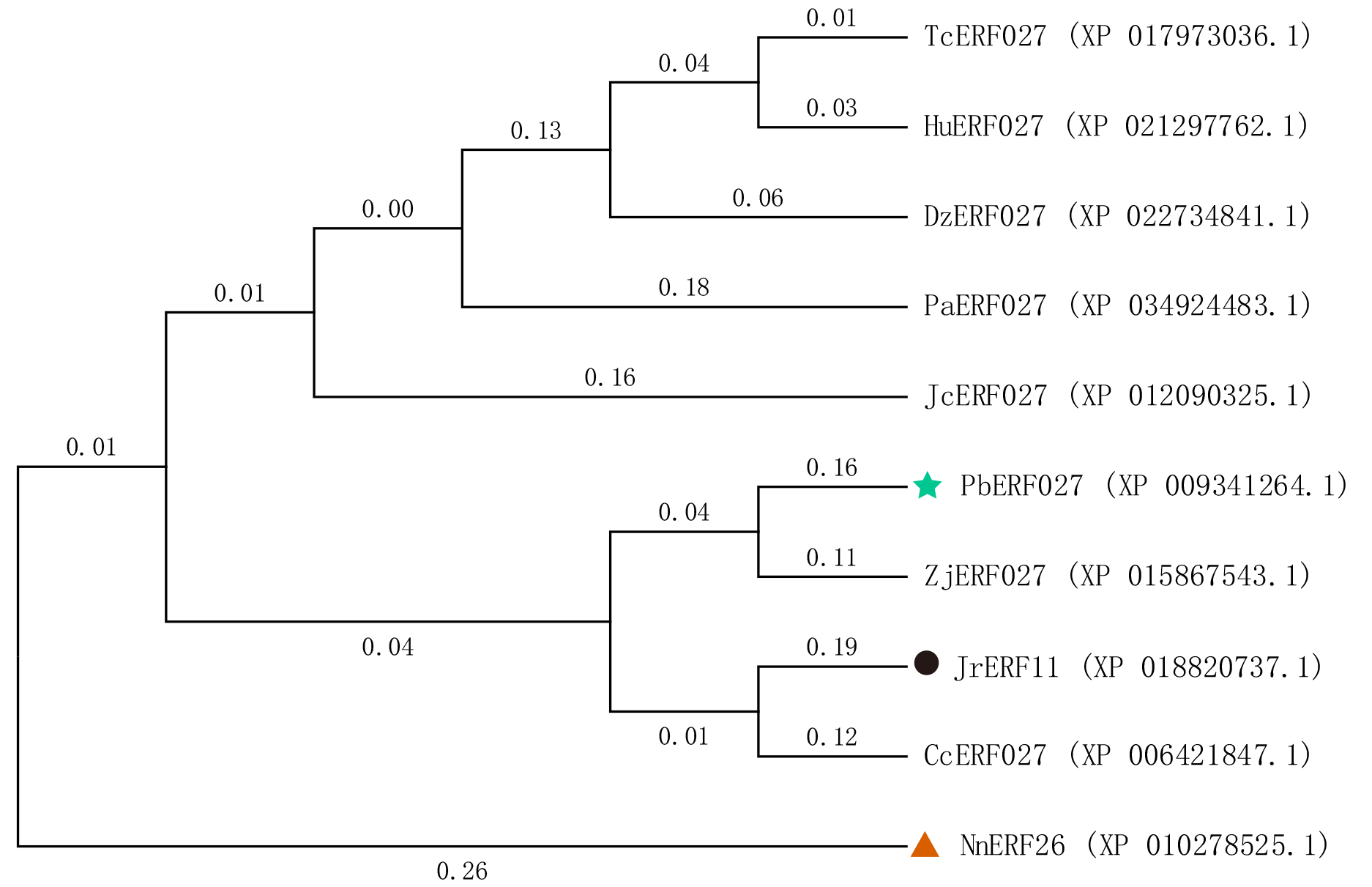

Supplement: Supplemental Information 3 — Tc: Theobroma cacao; Hu: Herrania umbratica; Dz: Durio zibethinus; Pa: populus alba; Jc: Jatropha curcas; Pb: Pyrus brestschneideri; Zj: Ziziphus jujuba; Jr: Juglans regia; Cc: Citrus clementina; Nn: Nelumbo nucifera. [file peerj-09-12429-s003.png]
