# Supplementary material for: Genome-wide identification and expression analysis of ethylene responsive factor family transcription factors in Juglans regia
Source: PeerJ. 2021 Nov 19;9:e12429. doi: 10.7717/peerj.12429 (PMC8607932; doi:10.7717/peerj.12429)
Supplement: Supplemental Information 4 [file peerj-09-12429-s004.docx]

Table S1 The primers for qRT-PCR analysis under different stresses and tissues

| Locus | The forward primer 5’~3’ | The reverse primer 5’~3’ |
| --- | --- | --- |
| JrERF01 | 5’-ATGTGTGGAGGAGCTATC-3’ | 5’-ATCGGCTTCAGACACGTT-3’ |
| JrERF02 | 5’-AGTGGAGCACGAGATCAT-3’ | 5’-GGATGTTCGAATTGCAGC-3’ |
| JrERF03 | 5’-ATGGTGAGCTTACGAAGG-3’ | 5’-AGATGAGCTCGAACCAGT-3’ |
| JrERF04 | 5’-TCCATGCACTCAACATGC-3’ | 5’-CGACATCATAAGCCTTGG-3’ |
| JrERF05 | 5’-TTGCTCACAGGTTGCCT-3’ | 5’-GTGTTGTAGGTACCGAG-3’ |
| JrERF06 | 5’-CGACCATCCCAATTGTTG-3’ | 5’-CACGCCTTGTTGAATCTC-3’ |
| JrERF07 | 5’-CAGAGTCGACTGTAGCAA-3’ | 5’-TGTCACCTGGTGTTGATG-3’ |
| JrERF08 | 5’-CCAGAACCTACTCATCAG-3’ | 5’-AAGCTCTAGCTGCCATCT-3’ |
| JrERF09 | 5’-ATGTGGCTAGTCCAGCAT-3’ | 5’-ATTGCACGTCCTCAGCACT-3’ |
| JrERF10 | 5’-ATACTGGTGCGAGCACAT-3’ | 5’-CGATCAACTGTGCATCGAT-3’ |
| JrERF11 | 5’-CTGTACAAGTACCTGAAGC-3’ | 5’-CGTACTCCTCGATACTTC-3’ |
| JrERF12 | 5’-AGCAGATCCTGGAGTGGTT-3’ | 5’-TCAGCCGAGTCAAACGTAC-3’ |
| JrERF13 | 5’-AGTCAAGCTCCAACTCGT-3’ | 5’-AGCTCCTCATTCGTACC-3’ |
| JrERF14 | 5’-TTCTGGAACTCCGACAG-3’ | 5’-ATGACTAGGCCATGGAG-3’ |
| JrERF15 | 5’-AGCATACAGGGAGGATTG-3’ | 5’-AAGTAGATGCCACGCTTG-3’ |
| JrERF16 | 5’-TGAAGGGTTCTCGAGCCAT-3’ | 5’-AGGAAGTCAAGAGCGTGT-3’ |
| JrERF17 | 5’-GAGTAAGGAAGAGACCGT-3’ | 5’-ACTCGTCGACTCAACGGT-3’ |
| JrERF18 | 5’AAGGATGAGGAAGTGGG-3’ | 5’-TCAGTCGCCTTCTTCCT-3’ |
| JrERF19 | 5’-ATGGTGAAGCAGACCACT-3’ | 5’-ATCTCCGTTGGAGTGAG-3’ |
| JrERF20 | 5’-CTAAGAAGTTCAGAGGCG-3’ | 5’-GGAATCCTCGCTAGTCTT-3’ |
| JrERF21 | 5’-GGTACTCAGTCTCAATCG-3’ | 5’-TTGGTCCTGGCCTTGATC-3’ |
| JrERF22 | 5’-ATCAGCATCAGCATCAGC-3’ | 5’-AGCAGCTGCAGCCATTCTT-3’ |
| JrERF23 | 5’-AGAGGTCCTTGAGAGATC-3’ | 5’-ATGGAGGGAGGAACGTAT-3’ |
| JrERF24 | 5’-CAACGCTAGATGAGGCTT-3’ | 5’-CACACATGACAAGTGTCC-3’ |
| JrERF25 | 5’-TCTGCTCTCACTCAGGTT-3’ | 5’-TGCATCCTCAGCTGTGT-3’ |
| JrERF26 | 5’-GCAGATCTCTACAGTAGC-3’ | 5’-GAAGTCGTAATCAGAAGG-3’ |
| JrERF27 | 5’-AACACCTTCTCGGAGACT-3’ | 5’-TCGAACCTTCAGCTGACT-3’ |
| JrERF28 | 5’-TGGCCAACGCAACAACAT-3’ | 5’-ATCCCTCTGTAGAGGTTC-3’ |
| JrERF29 | 5’-CATGTCACGACTAAGCTC-3’ | 5’-ATCTGGAATGCACTGCTG-3’ |
| JrERF30 | 5’-TTAGAGGAGTCAGGCAAC-3’ | 5’-TGCGAAGCTTGGTGCTAAG-3’ |
| JrERF31 | 5’-CTGAGATACGTGATCCCT-3’ | 5’-AAGAACTCACCGAGTACG-3’ |
| JrERF32 | 5’-GGCTCCGATAATGTGGAT-3’ | 5’-TTGGTTAGCTGCCGGCAT-3’ |
| JrERF33 | 5’-ATGGAGACAATCCCAGAC-3’ | 5’-CTGGAGAAGATCTCTCTG-3’ |
| JrERF34 | 5’-AGTCGTACGGATATCCGT-3’ | 5’-GGATACGTCGTAGAGGAT-3’ |
| JrERF35 | 5’-TGCAACATCGTCGACTAG-3’ | 5’-CACCGAATTCCACGATAC-3’ |
| JrERF36 | 5’-AATCCAGGCAATGAGCCAC-3’ | 5’-ACCATCGTTCATCGCGTAC-3’ |
| JrERF37 | 5’-AGCCATTCTCGACTCGAT-3’ | 5’-ATACCGGAGCAAGTGATG-3’ |
| JrERF38 | 5’-TCACCTACTTGGTGAGGT-3’ | 5’-TCGTTGTTCGAATGGTGG-3’ |
| JrERF39 | 5’-ATGGAAGAGACTAGAGGAGC-3’ | 5’-CTTCCCATCATCCCTACTCT-3’ |
| JrERF40 | 5’-TAACCAGGATTCCAGCTC-3’ | 5’-GAAGTTGGTCTTCGCCTT-3’ |
| JrERF41 | 5’-TCATCCAGGTGTAACCGT-3’ | 5’-CAGAGGCAGAAGTACTAG-3’ |
| JrERF42 | 5’-ATGGAAGGAGCTGCATGT-3’ | 5’-TCGTCATCGTCTTCTTGG-3’ |
| JrERF43 | 5’-CTTCGAGCGAAGATGAGT-3’ | 5’-GGTCTTCTACTGCTCGAT-3’ |
| JrERF44 | 5’-ATGATGAGGAGAGTCCGT-3’ | 5’-CTCCTCTGTACTTGGAAG-3’ |
| 18S rRNA | 5’-GGTCAATCTTCTCGTTCCCTT-3’ | 5’-TCGCATTTCGCTACGTTCTT-3’ |
